# Research on Preparation of Three-Component Composite Fiber with Complex Cross-Sectional Pattern

**DOI:** 10.3390/polym14112216

**Published:** 2022-05-30

**Authors:** Ronggen Zhang, Pei Feng, Chongchang Yang

**Affiliations:** 1 College of Mechanical Engineering, Donghua University, Shanghai 201620, China; rgzhang@mail.dhu.edu.cn (R.Z.); ycc@dhu.edu.cn (C.Y.); 2 Engineering Research Center of Advanced Textile Machinery, Donghua University, Shanghai 201620, China

**Keywords:** complex section patterns, three-component, composite fiber, numerical simulation, fiber preparation

## Abstract

In this work, a preparation method of three-component composite fibers with complex cross-sectional patterns was proposed, and the fibers with complex cross-sectional patterns were fabricated using melt spinning. Initially, inspired by the shape of a fishbone, a spinning pack with three-component melt channels was designed for spinning fibers with a “fishbone” cross-sectional pattern. Then, the numerical simulation of the melt flow in the channels of the spinning pack was performed using Polyflow software. The spinning pack structure was optimized by analyzing the flow velocity distribution and shear rate distribution of different components within the spinning pack channels. The results showed that smaller velocity fluctuations contribute to the clarity of the cross-sectional pattern. Thereafter, the spinning experiments were carried out based on the optimized spinning pack. The effect of the flow ratio between the three components on the cross-sectional pattern was discussed, and the three-component composite fibers with a clear “fishbone” cross-sectional pattern were obtained. Finally, in order to further study the effectiveness of the complex cross-sectional pattern fiber preparation proposed in this paper, another spinning pack for fibers with an “H-shaped” cross-sectional pattern was designed according to the aforementioned method, and spinning experiments were carried out. The SEM images of the cross-sections of fibers with “fishbone” and “H-shaped” cross-sectional patterns were obtained, verifying the feasibility of the method proposed in this paper. Moreover, the fibers with complex cross-sectional patterns obtained by this method have a certain anti-counterfeiting effect and can also be blended with other yarns to obtain fabrics with anti-counterfeiting effects.

## 1. Introduction

Composite spinning technology utilizes two or more polymers to prepare fibers with special properties and functions [1,2,3]. This spinning technology not only increases the diversity of fibers and their functions but could also reduce production costs [4,5].

During the preparation of multi-component composite fibers, the polymer melts of different components converge in the component, and the flow of components affects the cross-sectional state of the fiber [6,7]. When two or more polymer melts flow in the microporous flow channel, different microporous structure parameters and spinning process parameters cause differences in the flow velocity and pressure of the two polymer melts, which further affects different groups. The position of the interface and the shape of the fiber cross-sections were presented [8,9]. During the flow process, the material properties, the structural design of the spinning pack, and the spinning process have an effect on the fiber cross-sectional pattern. Multicomponent fibers with complex cross-sectional patterns are difficult and relatively expensive to imitate. Therefore, the fibers with complex cross-sectional patterns are processed together with other yarns to obtain textiles, and the authenticity of the textiles is judged by observing the cross-sectional shape of the yarns in the textiles using a microscope [10].

Currently, many scholars have carried out related research on the preparation of multi-component composite fibers. Rwei, et al. [11] comparatively studied the crimping properties of the composite fibers with different cross-sectional geometries, different types of fibers, and different spinning process conditions. Hwan, et al. [12] studied the effect of the tensile conditions on the properties of the composite fibers of different components. Kikutani, et al. [13] and Kohri, et al. [14] studied the spinning dynamic characteristics of the composite fibers based on the spinning dynamics theory of circular fibers. Radhakrishnan, et al. [15] studied the spinning dynamics of sheath-core composite fibers based on the assumption of uniform temperature distribution in the cross-section of the fiber. Zhang, et al. [16] developed a theoretical model for both melt blowing and electrospinning processes in order to explore the intrinsic curvature of the bicomponent fibers. The calculation results confirmed that a strain difference exists between the two components. Lund, et al. [17] demonstrated the melt spinning of a novel piezoelectric bicomponent polymer fiber using PVDF as an electroactive component. They manufactured a force sensor that consisted of several fibers embedded in a soft CB/CoPE elastomer matrix for piezoelectric characterization using an electrically conductive compound of CB. Further, HDPE was used as the core material and worked as an inner electrode. Matti, et al. [18] demonstrated the fiber spinning of anionic TEMPO-oxidized cellulose (TOCN) nanofibrils with polycations using interfacial polyelectrolyte complexation. The formed fibers were mostly composed of cellulose nanofibrils and the polycations were a minor constituent, leading to the yield and ultimate strengths of caprolactum were 100 MPa and 200 MPa, respectively; Young’s modulus of caprolactum was 15 GPa. This possibility was further exploited to demonstrate the reversible shape change of a bicomponent fiber directly by humidity change, and indirectly by temperature changes based on thermally dependent humidity absorption.

In addition to meeting the special functional requirements, the multi-component composite fiber with a complex cross-sectional pattern is prepared through the developed spinning pack, and the composite fiber is integrated into the structure of the product itself in order to achieve the anti-counterfeiting effect. Presently, the anti-counterfeiting fibers can be divided into dyeing, color-changing, fluorescent [19,20], textured [21], magnetic and composite fibers [22], and so on from the functional point of view of fibers. He, et al. [23] successfully prepared fluorescent and robust fibers with sodium alginate using the wet spinning of gold nanoclusters and alginate based on gold nanocluster-loaded alginate and studied the relationship between the process conditions, mechanical properties, and fluorescent properties of the fibers. Baatout, et al. [10] proposed a development method of fluorescent cotton yarn, and applied fluorescein into textile support using the impregnation method while keeping the original cotton yarn properties for anti-counterfeiting applications. Liu, et al. [24] studied the method of setting passwords using multi-bit sequence programming in the spinning process, and enabled passwords to be effectively stored throughout the fiber production and sales chain. Shen, et al. [25] developed a multifunctional optical fiber with quick-response reversible photochromic and light emission with a long afterglow using a wet spinning process, verified by experiments. It was found that the synthesized fiber had quick-response reversible photochromic properties. Zhang, et al. [26] prepared several kinds of spectrum-fingerprint fibers by using rare-earth strontium aluminate as the rare-earth luminescent material and fiber-forming polymers such as PET, PP, and PA6 as a matrix. Further, they were combined with transparent inorganic pigments and functional additives in order to study their emission spectra characteristics. The results showed that the emission spectral line of the spectrum fingerprint fiber is specific, similar to the fingerprint of a human being, which is hard to decipher and counterfeit and can be used to distinguish the original from the fake. Zhang, et al. [27] designed a skin-core structure and three kinds of dual-wavelength fluorescent anti-counterfeiting (DWFA) fibers, and excitable DWFA fibers with a skin-core structure. They were spun using fluorescent powder/PP pellets as the skin material and pure PP as the core material. The results showed that these DWFA fibers emitted two different colors of light under the excitation of two different wavelengths of light, which exhibited higher anti-counterfeiting safety. However, the process of product anti-counterfeiting in the above-mentioned documents is cumbersome and the cost is relatively high. Therefore, it cannot meet the anti-counterfeiting requirements of ordinary textiles.

In this work, a method to develop a three-component spinning pack with complex cross-sectional patterns was proposed. The flow velocity distribution of the three-component melt in the pores of the pack was simulated using Polyflow software. The numerical simulation of extrusion molding of three-component cross-section “fishbone” composite fibers shows the principle of patterning and forming three-component melts in components. Besides, by conducting spinning experiments and further adjustment of the preparation process, three-component composite fibers with a cross-sectional “fishbone” pattern were prepared, and the effect of the three-component flow ratio on the cross-sectional pattern was explored. Finally, the effectiveness of the method proposed in this paper was verified by preparing a cross-sectional “H-shaped” pattern three-component composite fiber, and the fibers had a good effect on anti-counterfeiting.

## 2. Numerical Simulation of the Three-Component Spinning Pack with Cross-Section “Fishbone” Pattern

The numerical simulation of the three-component spinning pack with a “fishbone” cross-section pattern includes the design and modeling of the spinning jack, spinning dynamic modeling, and the simulation parameter settings. Further, the results obtained during the simulation studies are discussed below.

### 2.1. Spinning Pack Design and Simulation Model

**Figure 1:**
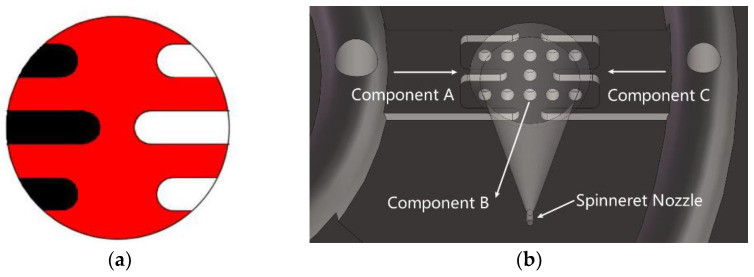
Design drawing of the cross section of the “fishbone” fiber. (**a**) The black domain is component A, red domain is component B, and white domain is component C. (**b**) The flow of polymers in the spinning pack.

**Figure 2:**
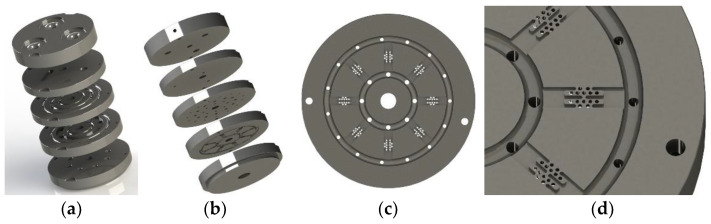
The 3D models of the spinning pack for the fiber with a “fishbone” cross-section. (**a**) Top view of spinning pack assembly; (**b**) bottom view of spinning pack assembly; (**c**) distribution board; (**d**) partial view of distribution plate.

Inspired by the shape of the fishbone, a “fishbone” fiber cross-section was designed as shown in Figure 1a. Figure 1b shows the flow of polymers of three components in the spinning pack. The black domain represents component A, the red domain is component B, and the white domain is component C. The spinning pack is the core component of the melt flow extrusion into the primary fiber during the spinning process. The designed three-component spinning pack with a cross-section “fishbone” pattern is shown in Figure 2.

During the design process of the spinning pack, it is necessary to consider the melt distribution of each distribution plate, the distribution form of the three components inside the pack, and the melt flow velocity and pressure difference of each component inside the pack caused by the complex structure, in order to avoid the influence of poor forming caused due to melt recombination and confusion in the cross-section pattern. Moreover, for the pack design of the three-component composite fiber, the flow consistency of the melts of different components in the spinning pack is the key to ensuring the clear cross-sectional pattern of the fiber.

### 2.2. Spinning Dynamic Model

In order to obtain a clear cross-section pattern, the dynamic characteristics of the melt flow in the channel of the cross-sectional “fishbone” pack were carried out using Polyflow software, and the flow velocity of the three melts while inside the pack and also entering the spinneret was studied.

In order to reduce the amount of calculation, the following assumptions were made for the polymer melt by considering the characteristics of the polymer melt and the process conditions for a stable extrusion.

Further, it was considered that the temperature change of the three melts could be ignored after entering the pack. Due to the high viscosity of the polymer melt, the Reynolds number is usually small while flowing, and in the spinning pack, the change in pressure and temperature has an insignificant effect on the density, and the melt density does not change in the flow. “Inertial force” and gravity can be ignored, and a small flow rate can be considered so that there is no slip between the melt and the wall of the runner.

The basic governing equations of the polymer melts flowing in the spinning pack lay a foundation for the distribution of velocity and temperature field in the process of the melt flow of different polymer materials. In the case of the spinning of composite fibers with any cross-sectional shape, assuming that there is no interfacial slip between different components, the continuity equation is expressed as shown in Equation (1).

(1) $$\frac{\partial \rho }{\partial t}+\rho \nabla \cdot u=0$$

In Equation (1), t refers to the time; ρ refers to the solution density; ∇ refers to the Hamilton differential operator; and $u=\left(u_{x},\;u_{y},\;u_{z}\right)$ is the velocity components along the direction of x, y, z, respectively.

For the spinning of the two polymer materials, assuming that the air friction force is negligible, the conservation equation of the spinning momentum of the composite fiber can be written as shown in Equation (2).

(2) $$\rho \left(\frac{\partial u}{\partial t}+u\cdot \nabla u\right)=\nabla \cdot \sigma +\rho g$$

In Equation (2), $\sigma =\left(\begin{array}{ccc} \sigma_{x} & \sigma_{y} & \sigma_{z} \end{array}\right)$ is the stress components along the direction of x, y, z, respectively; g refers to the acceleration of gravity.

For an isotropic fluid, the heat flux density is  q=−k∇T based on the Fourier heat conduction equation; then, the energy conservation equation of the flow field is expressed as shown in Equation (3).

(3) $$\rho C_{p}\frac{dT}{dt}=k\nabla \cdot \left(\nabla T\right)+\tau ∶\nabla u$$

In Equation (3), T refers to the temperature; $C_{p}$ refers to the isobaric specific heat; k refers to the thermal conductivity of the material; and τ refers to the stress tensor.

The Bird-Carreau model [28,29] is used to establish the constitutive equation of the melt, and its expression can be written as shown in Equation (4).

(4) $$\eta =\eta_{\infty }+\frac{\eta_{0}-\eta_{\infty }}{{\left[1+{\left(\lambda \dot{\gamma }\right)}^{2}\right]}^{\frac{1-n}{2}}}$$

In Equation (4), $\eta_{0}$ refers to the apparent viscosity; λ refers to the model parameter, that is, the relaxation time; n refers to the non-Newtonian exponents; $\eta_{\infty }$ refers to the shear limiting viscosity; and η refers to the melt shear viscosity.

Meanwhile, the zero-shear viscosity $\eta_{\infty }$, the apparent viscosity $\eta_{0}$, and the relaxation time λ depend on the temperature and follow the WLF equation as shown in Equation (5).

(5) $$\left\{\begin{array}{c} \eta_{0}\left(T\right)=a_{T}\eta_{0}\left(T_{0}\right)\\ \eta_{\infty }\left(T\right)=a_{T}\eta_{\infty }\left(T_{0}\right)\\ \lambda \left(T\right)=a_{T}\lambda \left(T_{0}\right) \end{array}\right.$$

In Equation (5), $\eta_{\infty }\left(T_{0}\right)$,$\;\eta_{0}\left(T_{0}\right),\;\mathrm{and}\;\lambda \left(T_{0}\right)$ denote the zero-shear viscosity, infinite shear viscosity and relaxation time at temperature $T_{0}$, respectively;$\;a_{T}$ refers to the conversion factor, and its value calculation formula satisfies the formula in Equation (6).

(6) $$lna_{T}=-\kappa \left(T-T_{0}\right)$$

In Equation (6), κ refers to the WLF equation parameters.

### 2.3. Simulation Parameter Settings

During the simulation and melt spinning of the three-component composite fiber in this paper, the material of component B is PA6 polymer, and both component A and component C are PP polymer. When simulating the melt flow using the Polyflow software, the micropore length is taken as 1.2 mm, the free section length is taken as 3 mm, and the room temperature is taken as 20 °C. Other parameters used in the simulation are listed in Table 1.

The melt inlet conditions are as follows: the inlet flow Q of the three components are set to be the same as 1.2 × $10^{-8}$ m^3^/s.

The runner wall condition is as follows: normal velocity Vn = tangential velocity Vτ = 0.

The melt outlet condition is as follows: fn = fs = 0 is set.

The Picard iterative algorithm is used to complete the iterative calculation for finite elements. This iterative algorithm is used to solve the equations with few unknowns, and the coupling between free surface iteration and calculation of velocity field and stress field is released.

The velocity fields are interpolated linearly by Mini-element, the 3D mesh is reset in the micro-hole part, and the slip coefficient is added to the holes boundary.

### 2.4. Simulation Results and Discussion

**Figure 3:**
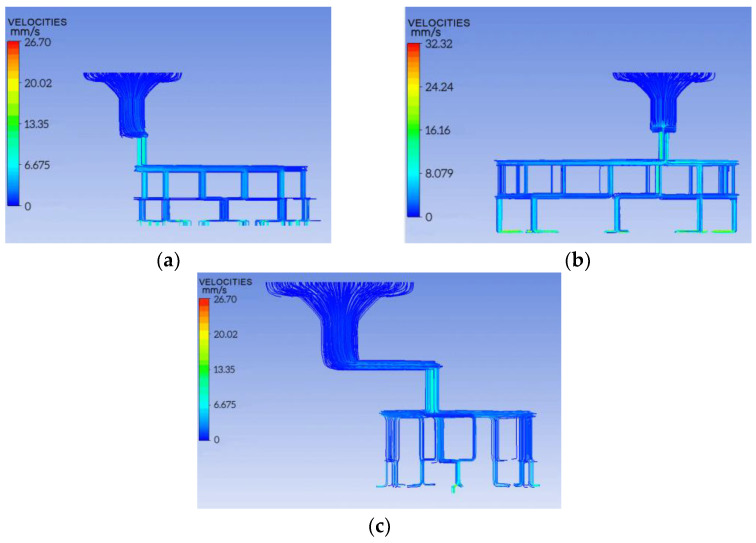
Velocity distribution of three components. (**a**) Component A; (**b**) component B; (**c**) component C.

The 3D software was used for modeling and imported into Polyflow software for numerical simulation, and the cloud map of the melt flow velocity in the channel of the three-component spinning pack with a cross-section “fishbone” was obtained, as shown in Figure 3.

From Figure 3, it is observed that the flow velocity of the three-component melt tends to change smoothly. Further, when the flow rate is constant, the flow velocity increases with the decrease in the pore diameter; the flow velocity of the melt near the wall is relatively small, and the maximum velocity is in the center of the channel. The maximum velocities of components A, B, and C are 26.70 mm/s, 32.32 mm/s, and 26.70 mm/s, respectively, and the maximum velocity difference of the three components is 5.62 mm/s. The small speed difference provides a good condition for the sharpening of the pattern section.

**Figure 4:**
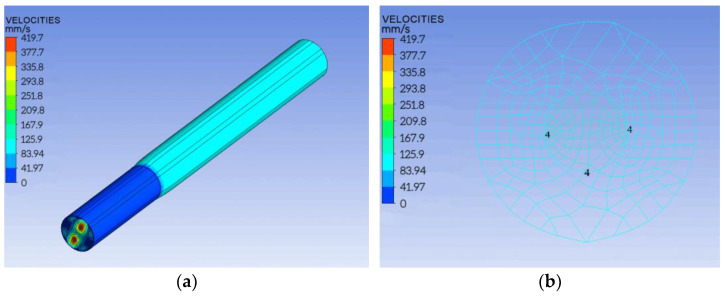
Three-component melt velocity distribution in the spinneret hole. (**a**) Overall cloud map; (**b**) outlet cloud map.

Figure 4a shows the cloud diagram of the melt flow velocity in the spinning pack of the three-component composite fiber. It is observed that the velocity of the melt is the largest at the entrance of the spinning guide hole, and the maximum velocity of component A and component C reaches 419.7 mm/s. Further, it is also observed that the velocity difference between components A and C is obvious, and the velocity of component B changes smoothly. Moreover, as the three components enter the spinneret micro-holes, and are extruded together, the velocity difference of components A and C gradually decrease. At 0.1 mm from the surface of the spinning pack, the speeds of the three components are found to be the same, as shown in Figure 4b.

**Figure 5:**
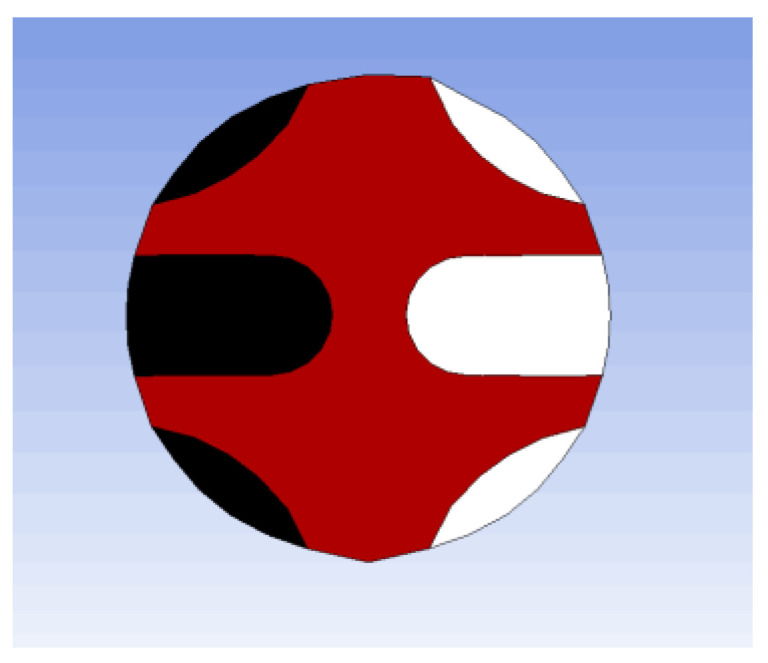
Simulation drawing of fiber with “fishbone“ cross-section.

From Figure 4, it is observed that after 0.1 mm from the spinneret, the cross-section “fishbone” pattern of the three-component composite fiber also tends to be stable because the velocity distribution is the same, as shown in Figure 5. From Figure 5, it is found that the different components of the cross-section of the composite fiber formed have clear contours, and the cross-section of the fiber presents a special “fishbone” pattern.

## 3. Preparation of Three-Component Composite Fibers

The fiber preparation equipment, materials, and the spinning process parameters that were used for the preparation of the three-component composite fibers are discussed below.

### 3.1. Fiber Preparation Equipment

**Figure 6:**
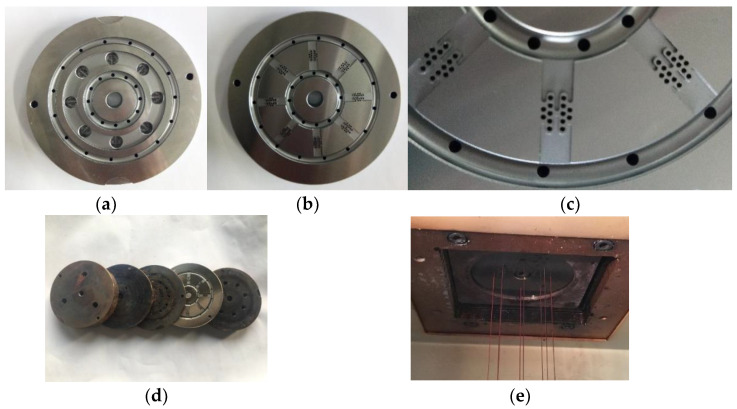
The spinning pack and equipment for the fiber with a “fishbone” cross-section. (**a**) Top view of the distribution board; (**b**) bottom view of the distribution board; (**c**) partial view of the distribution board; (**d**) the spinning pack assembly; (**e**) the spinning equipment.

A cross-sectional “fishbone” pattern three-component spinning pack and spinning equipment were developed, as shown in Figure 6. Initially, the pack was installed on the multi-component composite flexible spinning test platform. The three sets of screw spinning equipment have independent control modules, which can independently control the melt delivery temperature and flow speed in each component in order to achieve the required spinning requirements, and make the process adjustments. The spinning temperature was controlled using the temperature control system, so as to ensure that the viscosity of the three melts met the requirements of the spinning process.

### 3.2. Materials

The materials used in the melt spinning experiments are shown in Table 2. Among them, the supplier of PA6 was Guangdong Xinhui Meida Nylon Co., Ltd., Jiangmen, China. The supplier of PP was Shaoxing Yunxiang Chemical Fiber Co., Ltd., Shaoxing, China. Further, the supplier of masterbatch was Zhejiang Jincai New Material Co., Ltd., Haining, China.

### 3.3. Spinning Process Parameters

**Figure 7:**
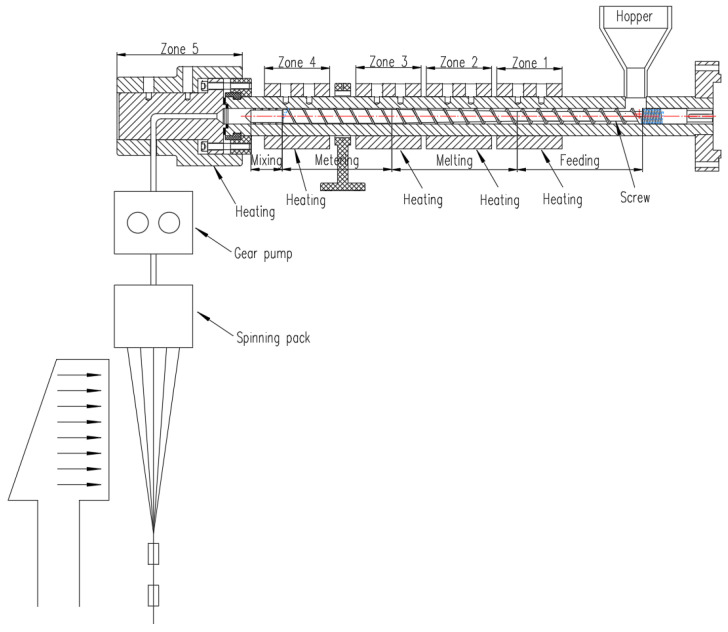
The schematic diagram of the melt spinning equipment.

Component A, component B, and component C were respectively transported to three screw extruders (manufacturer: Zhejiang Jinhu Group, Zhejiang Province, China. D = 30 mm, L/D = 25), and melted and extruded at a certain temperature. The extrusion of processed components included controlling the cooling conditions of the side blow setting to control its shaping. During the above process, the parameters of the spinning process that had to be controlled were the temperature of each zone of the screw, the temperature of the melt pipe, the temperature of the components, and the cooling wind speed. The total flow constant was maintained at 3.6 × $10^{-8}$ m^3^/s, so that the change in the flow of the three components and their flow ratios (component A: component B: component C) were 1:2:1, 3:4:3, 1:1:1, 4:3:4 and 2:1:2, respectively. The schematic diagram of melt spinning equipment that were used in the experiments and the temperature controller of each zone of the screw extruder are shown in Figure 7. The process parameters of the actual spinning experiment are shown in Table 3.

**Figure 8:**
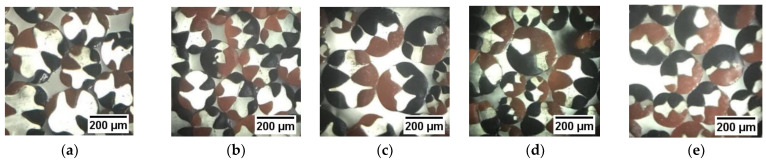
Cross-sections of “fishbone” fibers with different flow ratios. (**a**) The flow ratio is 1:2:1; (**b**) the flow ratio is 3:4:3; (**c**) the flow ratio is 1:1:1; (**d**) the flow ratio is 4:3:4; (**e**) the flow ratio is 2:1:2.

The spinning temperature parameters were individually set by the three-component spinning machine, and the flow ratio of the three components was controlled for fiber preparation. In order to obtain the experimental samples of the fiber to observe its cross-sections, a Y172 fiber slicer was used to slice the fiber, and the cross-section pattern of the fiber was observed using an optical microscope (model:BA210LED, manufacturer: Motic). Moreover, the fiber cross-sections obtained under different flow ratios of the three components are shown in Figure 8.

From Figure 8, it is observed that the red and black pattern shapes of the prepared three-component composite fiber cross-section are symmetrically distributed, and the “fishbone-shaped” cross-section pattern is clearly visible. It is observed from the research that the cross-sectional pattern of the “fishbone-shaped” three-component composite fiber changes with the change in the three-component flow ratio. Furthermore, as the flow of the red PP domain and the black PP domain becomes larger, the PA6 domain is gradually wrapped. When red PP:PA6: black PP = 2:1:2, the PA6 domain is found to be completely wrapped.

## 4. Application: Preparation of Three-Component Composite Fiber with an “H-Shaped” Cross-Section Pattern

**Figure 9:**
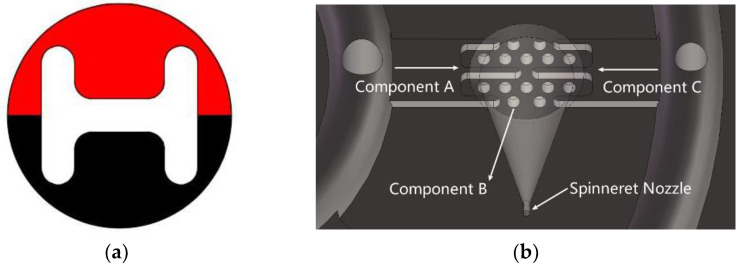
Design drawing of the cross-section of the “fishbone” fiber. (**a**) The red domain is component A, white domain is component B, and black domain is component C. (**b**) The flow of polymers in the spinning pack.

**Figure 10:**
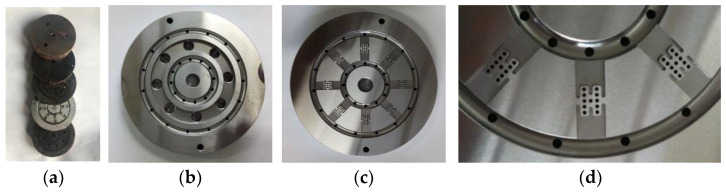
The spinning pack for the fiber with an “H-shaped” cross-section. (**a**) The spinning pack assembly; (**b**) top view of the distribution board; (**c**) bottom view of the distribution board; (**d**) partial view of the distribution board.

In order to further study the effectiveness of the fiber preparation with complex cross-section patterns proposed in this paper, an “H-shaped” fiber cross-section was designed as shown in Figure 9a. In Figure 9a, the red domain is component A, the white domain is component B, and the black domain is component C. Figure 9b shows the flow of the polymers of three components in the spinning pack. The “H-shaped” cross-sectional pattern three-component spinning assembly was designed and developed, and three-component composite spinning experiments were carried out. The designed “H-shaped” cross-sectional pattern three-component spinning assembly is shown in Figure 10.

**Figure 11:**
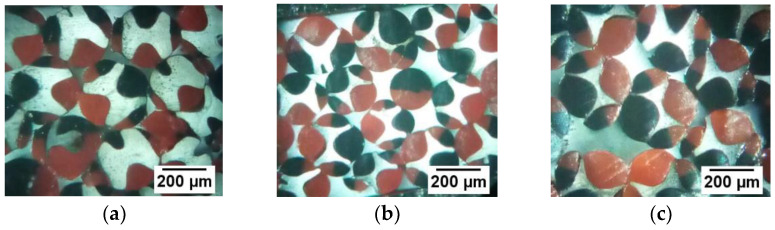
Cross-sections of “H-shaped” fibers with different flow ratios. (**a**) The flow ratio is 1:2:1; (**b**) the flow ratio is 1:1:1; (**c**) the flow ratio is 2:1:2.

The spinning process parameters of the “H-shaped” fiber are found to be the same as that of the “fishbone” fiber, as shown in Table 2. The total flow constant was maintained at 3.6 × $10^{-8}$ m^3^/s, so that the change in the flow of the three components and their flow ratios (component A: component B: component C) were 1:2:1, 1:1:1, and 2:1:2, respectively. Under the conditions of maintaining the total flow rate unchanged and changing the flow ratio of the three components, the “H-shaped” cross-sectional pattern three-component of the fiber was prepared. The cross-sectional diagram of the fiber was obtained using the same fiber slicing tool and an optical microscope as mentioned previously (Figure 11).

From Figure 11, it is observed that the cross-section of the three-component composite fiber has an “H-shaped” pattern formed by red PP and black PP wrapped with transparent PA6, and the cross-sectional pattern is clearly visible. The “H-shaped” pattern of the three-component composite fiber cross-section is found to vary with the flow ratio of the three components. As the flow of red PP and black PP becomes larger, the PA6 domain is gradually wrapped. When red PP: PA6: black PP = 2:1:2, the PA6 domain is completely wrapped.

**Figure 12:**
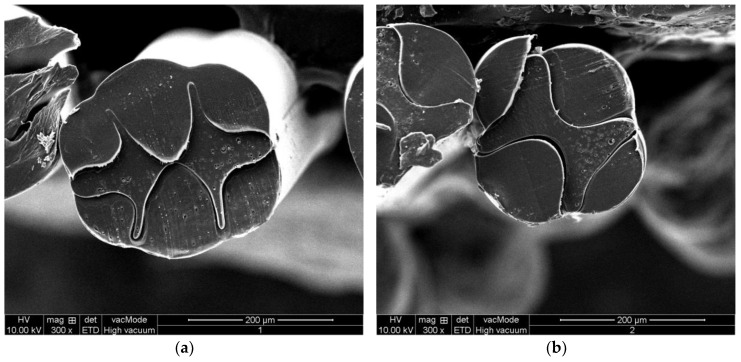
SEM image of the fiber cross-section. (**a**) “Fishbone” pattern; (**b**) “H-shaped” pattern.

In order to observe the fiber cross-section morphology more clearly, an electron microscope (model: JSM-5600LV, manufacturer: JEOL Ltd. Akishima-shi, Japan) was used, and its magnification ranged from 18 to 300,000 times. Several fibers were randomly selected from each group of fibers that were prepared in the experiment as samples. Before making the fiber section for SEM observation, in order to prevent the fiber section from being deformed due to cutting, the fibers were first frozen with liquid nitrogen, initially. Then, they were cut with a knife tool to obtain the section. Finally, according to the operating procedures, the fiber samples were placed under a scanning electron microscope for observation. The fiber cross-section SEM images of the optimized “fishbone” pattern three-component composite fiber and “H-shaped” pattern three-component composite fiber samples are shown in Figure 12.

It can be seen from Figure 11 that the shapes of the cross-sectional “fishbone” pattern three-component composite fiber are similar to that of the simulation results. Since PA6 and PP are immiscible, an obvious interface is produced. Further, the cross-section of the fiber prepared by the designed spinning pack is clear, the fiber cross-section changes regularly, and the cross-section shape of the fiber can be controlled to change regularly by changing the flow ratio. Furthermore, the product obtained by processing the fiber and other textiles together has a good anti-counterfeiting function. However, it is observed that there are some differences in the proportions of each component. The possible reason is that some conditions were simplified in the numerical simulation, such as setting the melt density constant with temperature and pressure, and insignificant penetration and slip between the two components. The constructing equations may also affect the results. Another reason may be that the parameters used for the simulation are not exactly the same as the actual parameters of the polymer melt. In the future, more numerical simulations and experimental results will be compared and tested in order to explore more complex cross-section fiber-forming laws.

## 5. Conclusions

Based on the basic principles of polymer rheology, a three-component spinning pack with a cross-section “fishbone” pattern was designed, and the numerical simulation of non-Newtonian fluids was carried out using Polyflow software. The flow mechanism of the melt in the three-component composite spinning package was explored. The flow velocity distribution diagram of different components in the pack was obtained, and the structure parameters of the spinning pack could be corrected using this method.

According to the governing equation of polymer rheology, the extrusion process of the cross-section “fishbone” three-component composite fiber in the spinneret hole was simulated using Polyflow software. The results showed that the three melts with different components have the highest velocity at the entrance of the spinneret guide hole and tend to be consistent with the melt extrusion. The extrusion velocity was the same at 0.1 mm from the spinneret surface, and the cross-section of the fiber became steady.

The multi-component composite flexible spinning platform was used for experimental preparation, and the effects of different spinning processes on the formation of fiber cross-section patterns were studied through experimental fiber preparation, especially the effect of flow ratio on the formation of fiber cross-sections. The cross-section patterns of the fiber prepared under different flow ratios of components were found to have a certain regularity. When the flow ratio of the three components was 3:4:3, there was a clear boundary on the cross-section of the fiber. Due to the particularity of the equipment and process for the preparation of cross-section “fishbone” and “H-shaped” patterned fibers, it was difficult to imitate and had a high anti-counterfeiting ability.

## Figures and Tables

**Table 1:** PA6 and PP material parameters and spinning process parameters.

Parameters	Component A	Component B	Component C
Non-Newtonian index n	0.35	0.82	0.35
Relaxation time s	0.003	0.02	0.003
Zero-shear viscosity Pa·s	115	750	115
$\mathrm{Density}\;\mathrm{kg}\cdot m^{-3}$	920	1135	920
$\mathrm{Thermal}\;\mathrm{conductivity}\;w\cdot m^{-1}k^{-1}$	0.18	0.2	0.18
$\mathrm{Isobaric}\;\mathrm{specific}\;\mathrm{heat}\;J\cdot \mathrm{kg}\cdot k^{-1}$	1700	3300	1700
Spinning temperature ℃	272	272	272
$\mathrm{Sin}\mathrm{gle}\;\mathrm{hole}\;\mathrm{inlet}\;\mathrm{flow}\;m^{3}\cdot s^{-1}$	$1.2\;\times \;10^{-8}$	$1.2\;\times \;10^{-8}$	$1.2\;\times \;10^{-8}$

**Table 2:** The materials used in the experiments.

	Material	Masterbatch
Component A	PP	Red
Component B	PA6	None
Component C	PP	Black

**Table 3:** Spinning process parameters of fiber with “fishbone” cross-sectional pattern.

Items		Component A	Component B	Component C
Screw extruder	Temperature of zone 1 (°C)	262	270	262
	Temperature of zone 2 (°C)	263	272	263
	Temperature of zone 3 (°C)	265	275	265
	Temperature of zone 4 (°C)	265	280	265
	Temperature of zone 5 (°C)	265	280	265
Temperature of gear pump (°C)		265	280	265
Temperature of spinning pack (°C)		280		
Humidity of cooling air (%)		65%RH ± 2%RH		
Speed of cooling air (m/s)		1.4		
Temperature of cooling air (°C)		15		
Room temperature (°C)		19		

## Data Availability

The data presented in this study are available on request from the corresponding author.
